# Surface‐electromyography characteristics of clonic seizures with no scalp‐EEG correlate: A comparative analysis with tremors

**DOI:** 10.1002/epd2.70035

**Published:** 2025-05-10

**Authors:** Veena V. Kumar, Akshaya R. Sivaji, Shwetank Singh, Zachary Scicchitano, Brandy Woods, Roohi Katyal, Neel Fotedar

**Affiliations:** ^1^ Epilepsy Center, Neurological Institute University Hospitals Cleveland Medical Center Cleveland Ohio USA; ^2^ Department of Neurology St. Luke's University Hospital Bethlehem Pennsylvania USA; ^3^ Clinical Translational Science Program Case Western Reserve University School of Medicine Cleveland Ohio USA; ^4^ Department of Neurology LSU Health Shreveport Shreveport Louisiana USA; ^5^ Department of Neurology Case Western Reserve University School of Medicine Cleveland Ohio USA

**Keywords:** clonic seizure, EEG, sEMG, tremor, Syndrome: focal non‐idiopathic parietal, Etiology: hemorrhage (cerebral), Phenomenology: clonic seizure, Localization: central (right), Syndrome: non epileptic paroxysmal disorder, Etiology: idiopathic, Phenomenology: clonic (non epileptic), Localization: not applicable

## Abstract

**Introduction:**

Clonic seizures are characterized by twitching movements at a frequency of 0.2–5 Hz. The clonic “twitch” is produced by a brief synchronized contraction of agonist and antagonist muscles, followed by a synchronized silent period. In this study, we aimed to compare the surface‐electromyography (sEMG) characteristics of scalp‐EEG negative clonic seizures with those of nonepileptic movements like tremors that can resemble clonic seizures.

**Methods:**

We retrospectively identified patients who were diagnosed with scalp‐EEG negative clonic seizures or tremors. We only included patients (*n* = 6) who were monitored simultaneously with video‐EEG and sEMG electrodes. sEMG was placed on agonist and antagonist muscles of the affected extremity using a standardized placement system developed at our institution. We analyzed the following characteristics of sEMG bursts: the relationship between agonist and antagonist muscles and the temporal evolution of burst duration, burst amplitude, and burst frequency.

**Results:**

The following sEMG characteristics were observed: (i) sEMG bursts and corresponding silent periods were synchronous between agonist and antagonist muscles in clonic seizures. In tremors, an alternating pattern was seen. (ii) sEMG burst amplitude increased during the first 10 s of clonic seizures. There was no significant change in tremors. (iii) sEMG burst duration increased from the beginning to end of clonic seizures. There was no significant change in tremors. (iv) sEMG burst frequency decreased from the beginning to end of clonic seizures due to increased burst and silent period duration. There was no consistent change in burst frequency in tremors. (v) sEMG burst duration of ≥250 ms was indicative of a clonic seizure with a >90% positive predictive value.

**Conclusions:**

Our study describes characteristic sEMG features of clonic seizures without scalp‐EEG correlates, which can be used as an objective biomarker in distinguishing these from nonepileptic movements such as tremors.

**Key points**

- Clonic seizures are characterized by synchronous sEMG bursts and silent periods of agonist and antagonist muscles.
- There is an increase in sEMG burst and silent period duration during a clonic seizure, leading to a slowing of the frequency of twitching.
- Clonic seizures without scalp‐EEG correlate are significantly shorter in duration compared with those with a correlate.

## 1 INTRODUCTION

Continuous EEG with simultaneous video monitoring is the gold standard for initial diagnosis and classification of seizures. Unfortunately, seizures might not have a scalp‐EEG correlate due to various reasons. Interpretation of scalp EEG can be limited by abundant artifact or technical error. Activation of at least 6 cm^2^ of cortical matter, and in most cases greater than 10 cm^2^, is required to produce a correlate on scalp EEG.^1^ The studies have shown that only 58% of seizures on intracranial EEG have a scalp‐EEG correlate. Of these, only 10%–33% of focal seizures with preserved awareness have a corresponding scalp correlate.^2^, ^3^ In these studies, seizures without a scalp correlate were significantly shorter in duration than those with a scalp correlate, at 49 s versus 89 s, respectively.^2^ This reflects the fact that focal seizures take time to recruit enough cortex to produce a scalp correlate. In these cases, and particularly with subtle symptomatology, there can be reasonable doubt in the classification of seizures. This poses a significant challenge to treatment when the diagnosis of epilepsy remains uncertain.

In this study, we aim to demonstrate the role of surface electromyography (sEMG) in the diagnosis of focal clonic seizures (focal aware motor (clonic)) with no scalp‐EEG correlate. sEMG measurements are noninvasive, technically easy, and high quality.^4^ Previous studies of sEMG have identified specific patterns of muscle activity unique to generalized tonic–clonic seizures (GTCs), which can reliably distinguish these from psychogenic nonepileptic events and acted seizures.^5^, ^6^

## 2 METHODS

We performed a retrospective analysis between 2021 and 2023 at our center to include consecutive patients who were diagnosed with scalp‐EEG negative clonic seizures or tremors. Inclusion criteria included the following: (1) Patients with a classification of seizure or tremor based on video‐recorded semiology by a board‐certified epileptologist (NF) and (2) patients who were monitored simultaneously with video‐EEG and sEMG. Exclusion criteria included the following: (1) significant background EMG signal or artifact hindering precise measurement of bursts, (2) any semiology (such as tonic) other than purely clonic or tremor, and (3) a lack of clear video for the duration of the event. EMG and EEG activity were recorded using Nihon Kohden QP‐112AK Ver. 10–03, JE‐921A 10–20 system amplifier, with the low pass filter set at 70 Hz and a sampling rate of 200 Hz. sEMG electrodes were placed on agonist and antagonist muscles of the affected extremities using a standardized placement system developed at our institution.^7^ Two electrodes were placed on each muscle, separated by 1 to 1.5 inches, and referenced to one another.

One typical motor event was analyzed for each patient. Of all the recorded events, we chose those with the clearest clonic twitching on video and cleanest EMG bursts for analysis. The EEG was then visually interpreted to confirm a lack of correlate for the motor event. Next, flexor and extensor sEMG channels were qualitatively compared to determine whether there was synchronous agonist and antagonist muscle activation, and whether muscle activation was followed by complete relaxation (termed the silent period). Finally, either the flexor or extensor sEMG channel was analyzed for the following characteristics for each second in time: burst duration, burst amplitude, and burst frequency. We chose between the flexor and extensor channels based on which had the highest amplitude, and thus the most robust signal. This decision was mainly qualitative based on the visual analysis of the EMG signal.

We included the measurements for each second in time for the duration of the clinical event, starting at the clinical onset and ending at the clinical offset on video. Multiple sEMG burst measurements within each second were averaged to produce a single burst duration, amplitude, and frequency measurement per second. There were between one and four measurement points averaged per second. Calculations were made manually using the Nihon‐Kohden time and voltage cursor tool, on a 5‐s page, with a sensitivity of 5 μV/mm and a time constant of .003 s, and with the 60 Hz filter on. Burst duration was measured by placing the first measurement cursor at the start of each burst and a second measurement cursor at the end of each burst. Burst frequency was measured by placing the first measurement cursor at the start of one burst and a second measurement cursor at the start of the next burst. Burst amplitude was measured by placing the first measurement cursor at the highest visible peak below the baseline and a second measurement cursor at the highest visible peak above the baseline, within each burst (Figure S1). We plotted these numbers against time using a scatterplot. By choosing the cleanest EMG bursts and by having the same data analyst measure each event at least twice to reduce human error, we ensured that our data would be reproducible. However, since the placement of the cursor is a manual process, there could be inter‐examiner variability, which is a limitation of this method.

Back‐averaging was performed to identify potential preceding discharges in scalp negative clonic seizures for patient 1. We used the online available platform *BacAv* ^8^ for this purpose. We identified 50 consecutive sEMG bursts and extracted the EEG and EMG data as .txt files to upload to the *BacAV* platform. We used C4‐A1 as the EEG channel and the flexor EMG channel for analysis.

To compare variables between groups, the Mann–Whitney test was used for analysis.^9^ A *p*‐value of <.05 was considered statistically significant.

The study was approved by the Institutional Review Board at University Hospitals of Cleveland.

## 3 RESULTS

Three patients with EEG negative clonic seizures and three patients with tremors were studied (Table 1).

**TABLE 1:** Patient demographics.

Patient	Age (year)	Sex	Location	Event	Etiology	Classification
1	68	M	ICU	Left hand twitching	Subdural hematoma	Clonic seizure
2	77	M	Floor	Right hand twitching	Metastatic melanoma	Clonic seizure
3	65	F	ICU	Right arm twitching	Meningioma	Clonic seizure
4	69	M	ICU	Left hand twitching	Traumatic brain injury	Tremor (2–5 Hz)
5	48	M	AMU	Right hand twitching	Idiopathic	Tremor (2–3 Hz)
6	30	F	ICU	Right hand twitching	Subarachnoid hemorrhage	Tremor (5–8 Hz)

Abbreviations: AMU, Adult Epilepsy Monitoring Unit; ICU, intensive care unit.

Clinically, all the events analyzed involved hand or arm twitching. The events clinically classified as clonic seizures were characterized by “twitching,” whereas the tremors had a “sinusoidal” character (Videos 1 and 2). All three tremors were undifferentiated, however, patients 4 and 5 had low frequency, low amplitude tremors that resembled parkinsonian tremors. Patient 6's tremor was higher in frequency and amplitude.

**VIDEO 1:** Clonic seizure. Left hand clonic seizure (“twitching” character) in patient #1 with simultaneous sEMG tracings for left forearm flexor (blue channel) and forearm extensor (pink channel) muscles showing synchronous bursts and silent periods. Video content can be viewed at https://onlinelibrary.wiley.com/doi/10.1002/epd2.70035

**VIDEO 2:** Tremor. Right hand tremor (“sinusoidal” character) in patient #5 with simultaneous sEMG tracings for right forearm flexor (pink channel) and forearm extensor (green channel) muscles showing alternating bursts and silent periods. Video content can be viewed at https://onlinelibrary.wiley.com/doi/10.1002/epd2.70035

**FIGURE 1:**
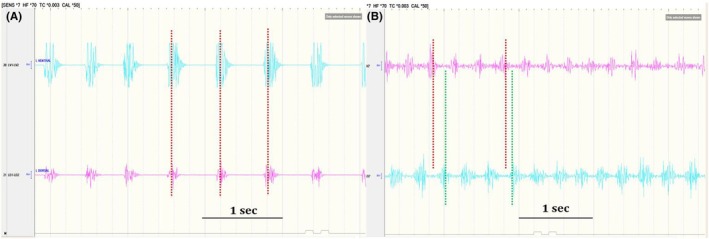
sEMG characteristics of agonists and antagonists. (A) Synchronous agonist and antagonist sEMG bursts and corresponding silent periods (red dotted lines) in clonic seizure. (B) Alternating agonist and antagonist sEMG bursts (red and green dotted lines) in tremor. Top sEMG channel‐forearm flexor compartment. Bottom sEMG channel‐forearm extensor compartment. sEMG, surface‐electromyography.

The sEMG bursts and corresponding silent periods were always synchronous between agonists and antagonists in clonic seizures (Figure 1A), as opposed to tremors, where an alternating pattern was seen (Figure 1B).

**FIGURE 2:**
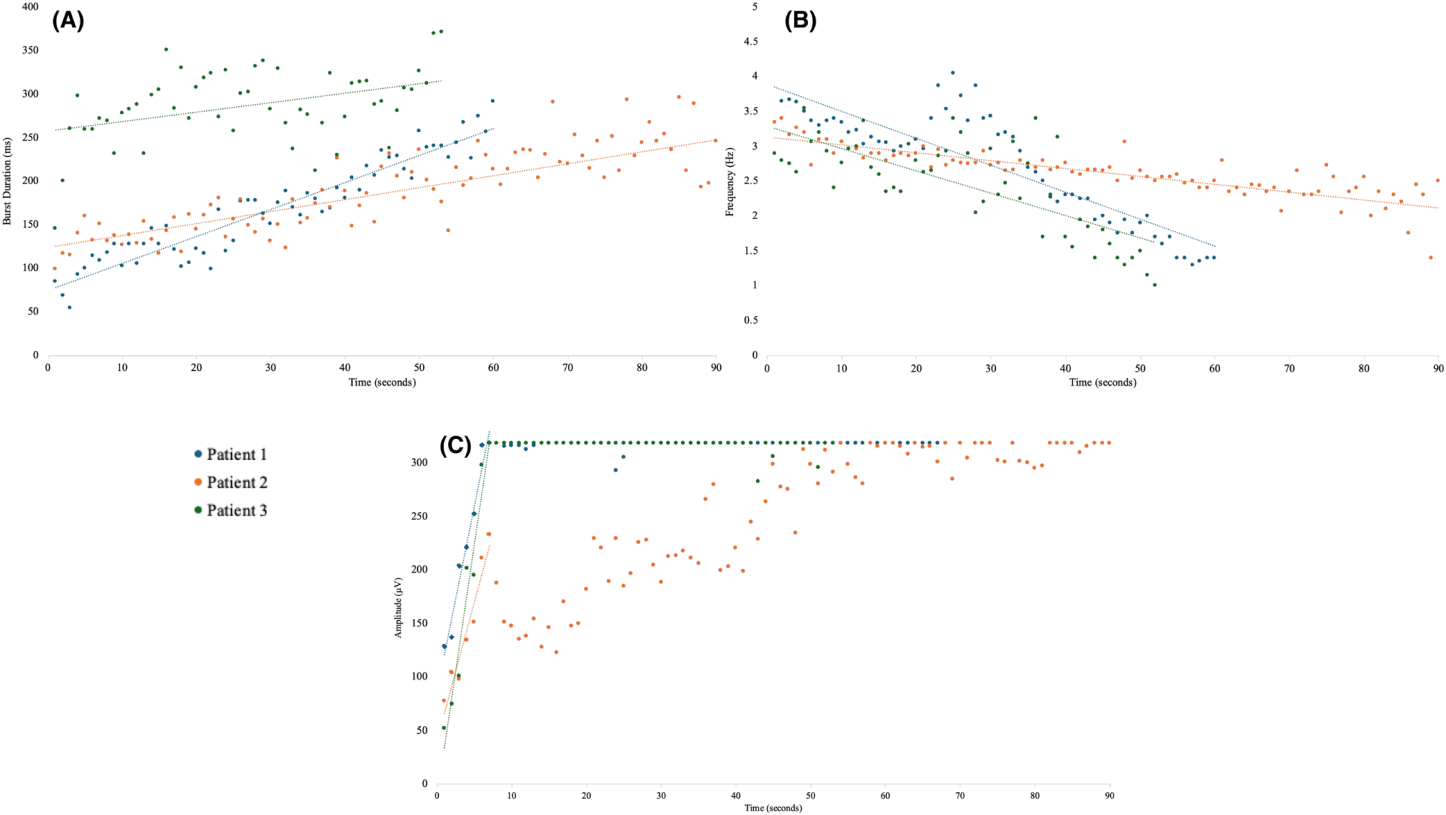
Scatterplot demonstrating temporal evolution of sEMG bursts in clonic seizures. (A) Linear increase in average burst duration over 60 s (dotted lines). (B) Linear decrease in burst frequency (dotted lines). (C) Linear increase in burst amplitude. The amplitude reaches a crescendo at ~10 s and then saturates the amplifier (dotted lines). sEMG, surface‐electromyography.

**FIGURE 3:**
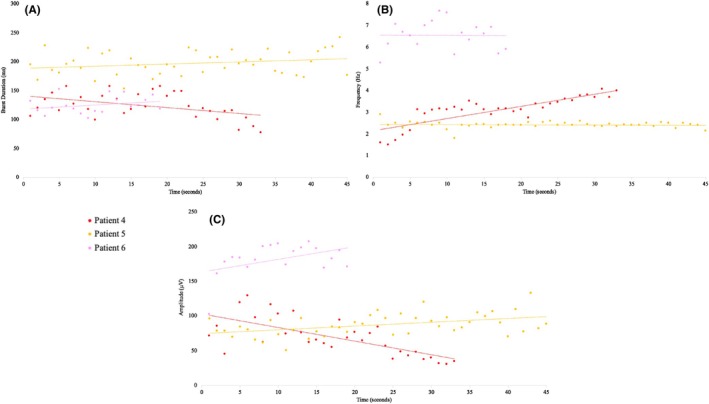
Scatterplot demonstrating temporal evolution of sEMG bursts in tremors. (A) No specific temporal evolution of burst duration. (B) No specific temporal evolution of burst frequency. (C) No specific temporal evolution of burst amplitude. sEMG, surface‐electromyography.

**FIGURE 4:**
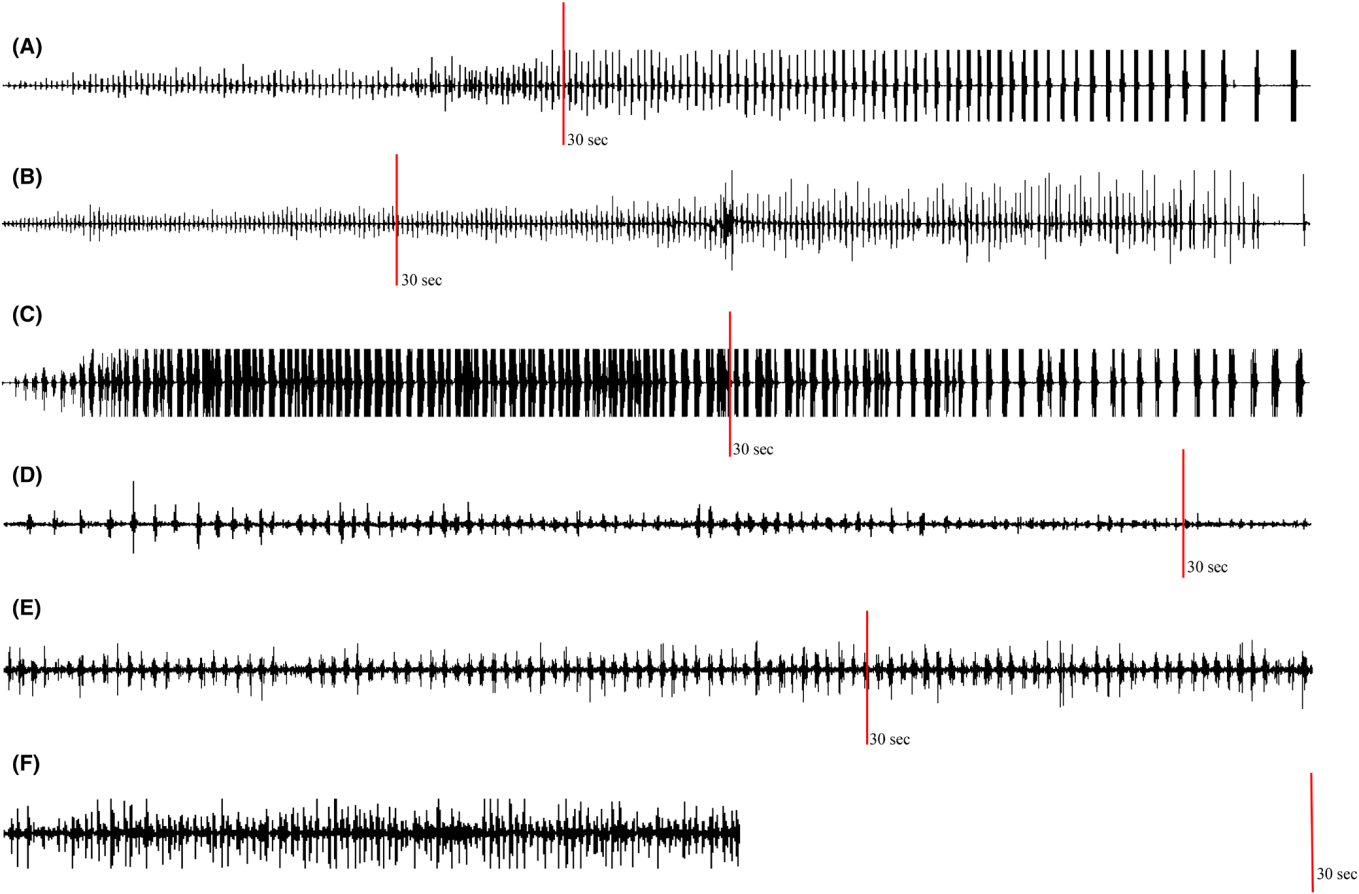
Raw sEMG tracings for clonic seizures and tremors. Red lines represent the 30‐s mark, for scaling purposes. (A–C) Clonic seizures. There is an increase in burst and silent period duration over time, with a clear decline in burst frequency consequently. (D–F) Tremors. There is no consistent change in burst amplitude, duration, or frequency over time. sEMG, surface‐electromyography.

In clonic seizures, a linear increase in burst duration was seen, along with a linear decrease in burst frequency over time (Figure 2A,B). No consistent change was noted in tremors (Figure 3A,B). There was also a consistent increase in silent period duration seen in clonic seizures, which was not seen in tremors and was the main contributor to the decrease in burst frequency over time (Figure 4A–F).

There was a linear increase in burst amplitude within the first 10 s of the seizure, after which point the amplifier was saturated in patients 1 and 2 (Figure 2A–C, Figure 4A–C). In contrast, in tremors, there was no consistent change in burst duration, amplitude, or frequency over time (Figures 3A–C and 4D–F).

**FIGURE 5:**
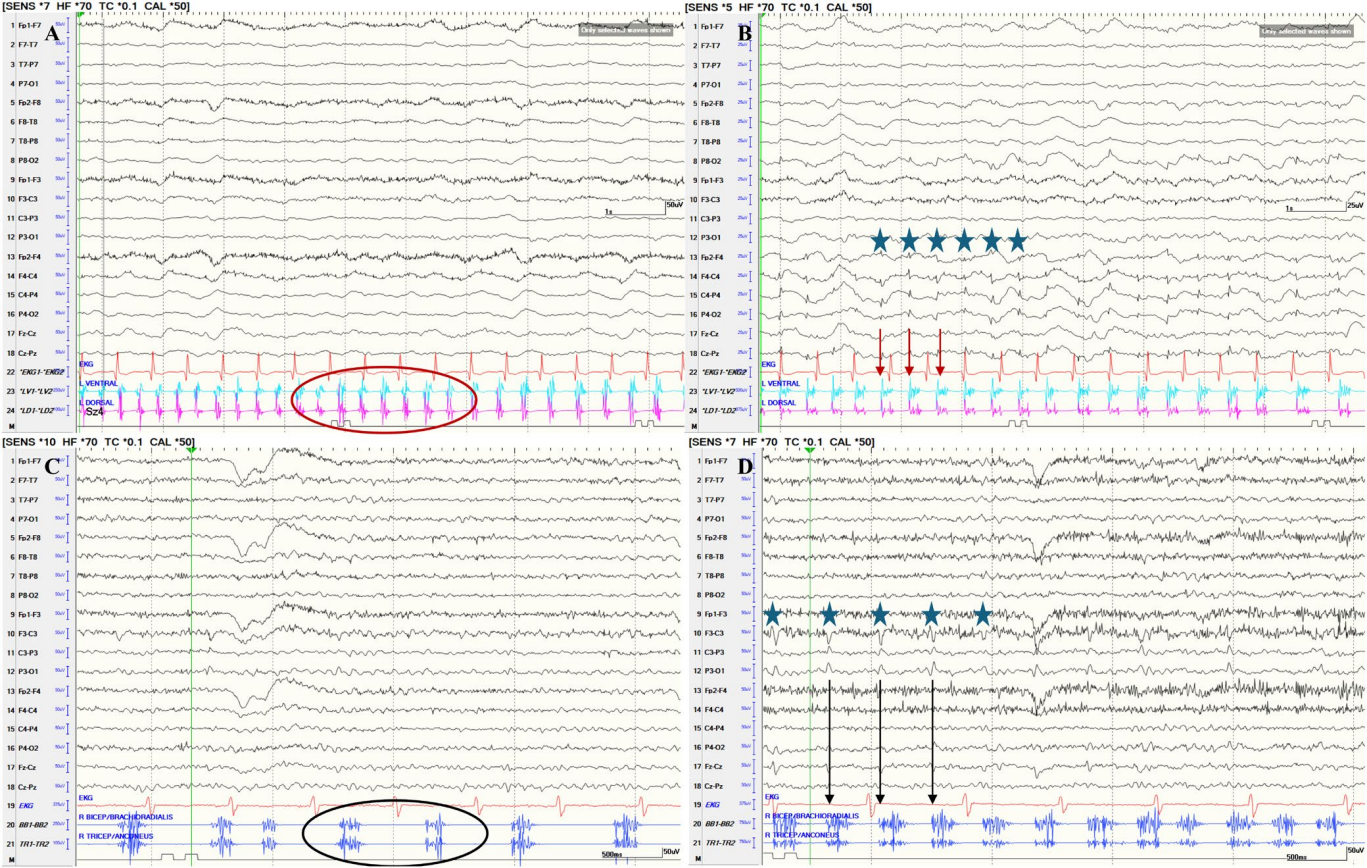
EEG‐sEMG for patients #1 and #3 demonstrating clonic seizures with and without EEG correlate. (A) 10s page showing a clonic seizure without EEG correlate for patient #1. Agonist and antagonist sEMG channels (bottom two channels) show discrete and synchronous bursts and silent periods (red circle). (B) 10s page showing a clonic seizure with EEG correlate for patient #1. Agonist and antagonist sEMG channels (bottom two channels) show discrete and synchronous bursts (red arrows) time‐locked to periodic spikes (highlighted by blue stars) localized to the right peri‐rolandic region. (C) 5 s page showing a clonic seizure without EEG correlate for patient #3. Agonist and antagonist sEMG channels (bottom two channels) show discrete and synchronous bursts and silent periods (black circle). (D) 5 s page showing a clonic seizure with EEG correlate for patient #3. Agonist and antagonist sEMG channels (bottom two channels) show discrete and synchronous bursts (black arrows) time‐locked to periodic spikes (blue arrows) localized to the left peri‐rolandic region. EEG is shown in a longitudinal bipolar montage (left temporal, right temporal, left parasagittal, right parasagittal and midline). sEMG, surface‐electromyography.

For patients 1 and 3 classified with clonic seizures, there were other similar events recorded on video‐EEG with a clear scalp‐EEG correlate supporting the diagnosis of epilepsy (Figure 5). In addition, for patient 1, we were able to identify a potential preceding the sEMG burst by back‐averaging 50 consecutive bursts. The EEG demonstrated a biphasic potential with initial positivity at C4. This potential had the same morphology as the one recorded in seizures with a clear scalp‐EEG correlate, thus localizing the generator to the peri‐rolandic cortex (Figure S2). The EEG‐sEMG latency was <20 ms, consistent with corticospinal tract conduction for the upper extremity. The sEMG characteristics of seizures with and without EEG correlate were identical. The sEMG bursts and corresponding silent periods were synchronous between agonist and antagonist muscles in seizures with a clear EEG correlate as well (Figure 5B,D). In addition, we also analyzed the temporal evolution of sEMG burst duration, frequency, and amplitude in seizures with EEG correlate, and the pattern was similar to the seizures without EEG correlate (Figure S3). Patient 2 did not have any seizures with a clear scalp‐EEG correlate; however, this patient had clear evidence of focal continuous slowing in the left hemisphere and evidence of metastatic lesions. In addition, the patient responded dramatically to anti‐seizure medications and the seizures stopped within 24 h.

We analyzed the duration of 10 consecutive seizures with and without scalp‐EEG correlate, each. For patient 1, the mean duration of seizures with scalp‐EEG correlate was 95.3 ± 28.8 s (95% CI 77.5–113.2 s) and that for seizures without scalp‐EEG correlate was 63.7 ± 8.5 s (95% CI 58.4–68.9 s). Using the Mann–Whitney test, this difference was statistically significant with *p* = .008. For patient 3, the mean duration of seizures with scalp‐EEG correlate was 61.5 ± 12.7 s (95% CI 53.6–69.3 s) and that for seizures without scalp‐EEG correlate was 42.5 ± 9.1 s (95% CI 36.9–48.1 s). Using the Mann–Whitney test, this difference was statistically significant with *p* = .002.

**FIGURE 6:**
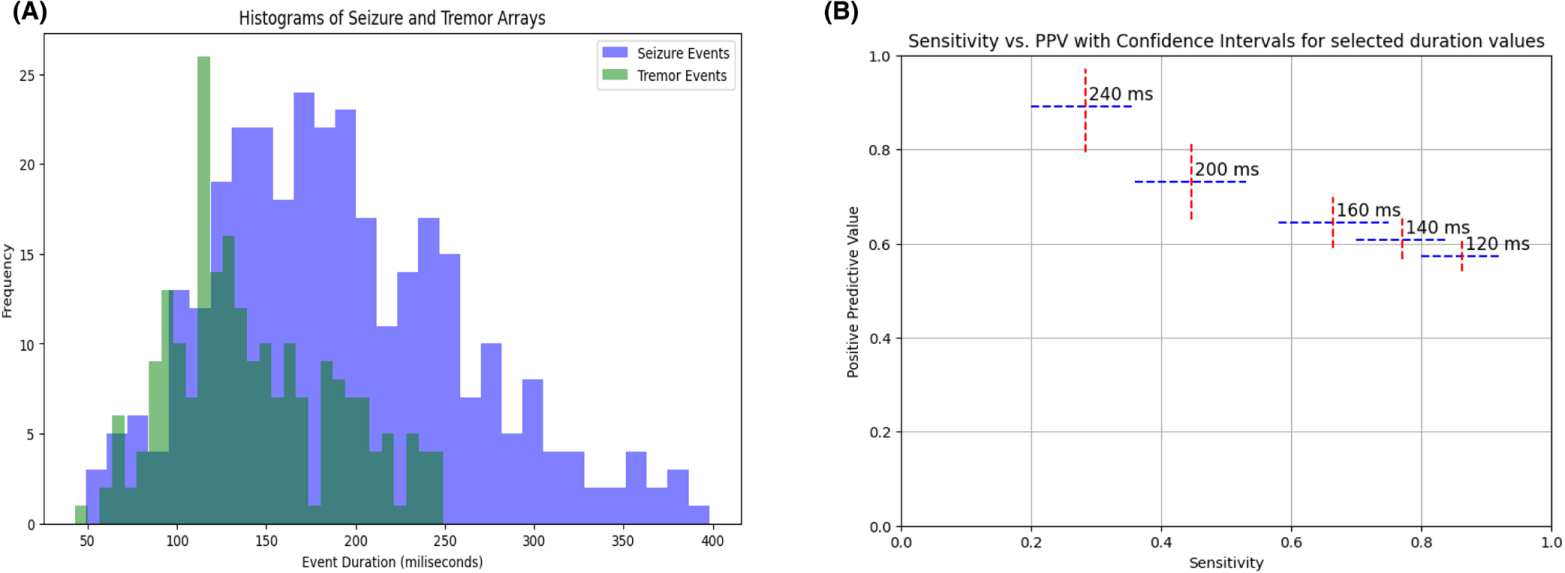
Statistical comparison of sEMG burst duration for clonic seizure and tremor. (A) Two overlapping histograms representing the sEMG burst durations of clonic seizure and tremor events, with no tremor burst observed lasting beyond 250 ms. (B) Sensitivity on the *x*‐axis and positive predictive value on the *y*‐axis for various burst duration cutoff values differentiating clonic seizures from tremors, with cutoff burst durations labeled (in ms). The PPV of the sEMG burst duration for clonic seizure over tremor is >90% for a burst duration of ≥250 ms. PPV, positive predictive value; sEMG, surface‐electromyography.

Using the Mann–Whitney test, we found a statistically significant difference in the sEMG burst duration between clonic seizures and tremors (51.8 ms, 95% CI 51.6–52.0 ms) (*p* = .0013). The mean sEMG burst duration for clonic seizures was 200.1 ms, and the mean sEMG burst duration for tremors was 148.3 ms. To provide a clearer direction for interpretation, we aimed to establish a specific time frame that could accurately distinguish a clonic seizure from a tremor. We randomly identified 64 bursts in each group (clonic seizure and tremor). We used overlapping histograms to compare the sEMG burst durations of clonic seizures and tremors. sEMG burst duration of ≥250 ms could reliably distinguish between clonic seizure and tremor with a positive predictive value (PPV) of >90% (Figure 6). Because some sEMG bursts in clonic seizures can have burst duration less than 250 ms, they may be misclassified as tremor events using this cutoff, reducing the sensitivity. The sEMG burst duration, in isolation, must not be used as a criterion to distinguish between clonic seizure and tremor. The other qualitative characteristics described here, in conjunction with the sEMG burst duration, together can help clinicians distinguish between these movements. The corresponding sensitivities and positive predictive values for sEMG burst duration cutoffs are shown in Table S1.

## 4 DISCUSSION

Clonic seizures are characterized by repetitive twitching movements at a frequency of 0.2 to 5 Hz.^10^ Focal clonus resembling a focal clonic seizure can be reproduced by direct electrical stimulation of the primary motor area (M1) in the precentral gyrus.^11^, ^12^ The existing literature on sEMG patterns of focal clonic seizures is relatively sparse. One study showed that raw and processed sEMG data analyzed by epileptologists who were blinded to the video‐EEG accurately classified 89% of tonic–clonic and tonic seizures, but only 33% of clonic seizures.^13^ In this study, we identified characteristic sEMG findings in focal clonic seizures with no scalp‐EEG correlate, like the ones reported by our group in a previous publication.^7^ We also compared these findings with those of tremors.

We observed a high concordance among the EMG tracings of the three patients with clonic seizures, despite a wide variability in demographics, acuity, event length, and etiology. Clonic seizures always showed a temporal evolution that was clear on sEMG but difficult to quantify visually at the bedside. Tremors, in contrast, showed random variability without reliable changes over time.

During clonic seizures without EEG correlate, there were always synchronous sEMG bursts of agonist and antagonist muscles alternating with synchronous silent periods. This is consistent with previous studies of focal clonic seizures with clear EEG correlate, which have shown synchronous activation and relaxation of agonists and antagonists.^7^, ^8^ In addition, we confirmed this observation in this study as well for seizures with a clear EEG correlate in patients #1 and #3. In contrast, tremors always had an alternating pattern in our patients. Few previous studies have also shown an alternating pattern of activation of agonists and antagonists in parkinsonian, Holmes, and functional tremors.^14^, ^15^, ^16^, ^17^

In addition, clonic seizures demonstrated a consistent rapid increase in sEMG burst amplitude and duration with a corresponding decrease in burst frequency from the start to end of clonic seizures (Figures 2A–C and 4A–C). We have reported these characteristics in our previous studies on spontaneous clonic seizures with a clear EEG correlate.^7^ We have observed almost identical characteristics in clonic twitching induced by electrical stimulation of the primary motor cortex.^12^ During high frequency stimulation (≥20 Hz), type II clonic responses are obtained (defined by wide EMG bursts >50 ms). As the stimulation train continues, there is a progressive increase in sEMG burst amplitude and duration, likely produced by an earlier summation of excitatory post‐synaptic potentials, because of their shorter duration (25–50 ms). The progressive decrease in sEMG burst frequency is due to a combination of prolonged EMG bursts and prolonged silent periods, which is likely related to later summation of inhibitory post‐synaptic potentials, because of their longer duration (>100 ms), mediated by recurrent pyramidal tract GABAergic collaterals.^12^

Tremors, in contrast, show random variability over time, likely because there is no summation effect of synchronized electrical activity (Figures 3A–C and 4D–F). It is postulated that tremors are produced by neuronal coupling between different groups of neurons that can produce sustained oscillations. The oscillatory behavior is driven by the phenomenon of post‐inhibitory rebound. The two groups of neurons are activated in an alternating manner and hence activate different groups of muscles in an alternating manner.^18^ This alternating activation and relaxation of agonist and antagonist muscles give tremors their “sinusoidal” appearance.

These key differences between clonic seizures and tremors are summarized in Table 2.

**TABLE 2:** Key clinical and sEMG findings of clonic seizures and tremors.

Characteristics	Clonic seizure	Tremor
Clinical	‘Twitching’ appearance because of fast twitch of both agonist and antagonist muscles, followed by slow relaxation	‘Sinusoidal’ appearance because of alternating contraction and relaxation of agonist and antagonist muscles
sEMG	Synchronous bursts and silent periods of agonist and antagonist muscles	Alternating bursts and silent periods of agonist and antagonist muscles
	Linear increase in burst and silent period durations from beginning to end of seizure	No consistent change in burst and silent period durations
	Linear decrease in burst frequency from beginning to end of seizure	No consistent change in burst frequency
	Linear increase in burst amplitude	No consistent change in burst amplitude
	Mean burst duration 200.1 ms	Mean burst duration 148.3 ms

Abbreviation: sEMG, surface‐electromyography.

Our study has some limitations, including a small sample size of six patients. This was due to the logistical difficulty in finding patients with EEG‐negative seizures and tremors who were simultaneously monitored with sEMG. The characteristics of both groups were different, with younger patients in the tremor subgroup. We would posit, however, that the characteristics of the EMG bursts of clonic seizures would not be expected to change significantly with age. However, larger studies would be required to verify this. By including patients in the intensive care unit, some tremors or seizures may have been influenced by metabolic derangements or sedative drug effects. Our sampling rate for sEMG was 200 Hz, which is lower than the standard 1000 Hz, likely resulting in a lower quality signal. While the tremors included in our study were undifferentiated, two of the three appeared most similar to a parkinsonian tremor. It is possible that different underlying etiologies of tremor could produce different EMG characteristics. Our results thus cannot be extrapolated to other types of tremors and nonepileptic movements. Further research would be required to compare sEMG characteristics of clonic seizures and other nonepileptic movements.

## 5 CONCLUSION

Surface electromyography can provide objective and reliable information to aid in differentiating clonic seizures without scalp‐EEG correlate from tremors.

**Test yourself**

1. Which of the following sEMG features is typical of clonic seizures?
  - Alternating sEMG bursts of agonist and antagonist muscles
  - Isolated extensor sEMG bursts
  - Isolated flexor sEMG bursts
  - Synchronous flexor and extensor sEMG bursts
2. Which of the following features is characteristic of clonic seizures as opposed to tremors?
  - Linear increase in sEMG burst amplitude over time
  - Linear decrease in sEMG burst frequency over time
  - Linear increase in sEMG burst duration over time
  - All of the above
3. Which of the following is true regarding differences between clonic seizures with and without scalp‐EEG correlate?
  - They have different sEMG characteristics
  - Seizures with scalp‐EEG correlate are longer than those without
  - Seizures with scalp‐EEG correlate are shorter than those without
  - None of the above

*Answers may be found in the* *supporting information*.

## Supporting information


Figure S1.

Figure S2.

Figure S3.

Table S1.

Appendix S1.

## Data Availability

The data that support the findings of this study are available on request from the corresponding author. The data are not publicly available due to privacy or ethical restrictions.
