# Supplementary material for: Surface‐electromyography characteristics of clonic seizures with no scalp‐EEG correlate: A comparative analysis with tremors
Source: Epileptic Disord. 2025 May 10;27(4):609–19. doi: 10.1002/epd2.70035 (PMC12398199; doi:10.1002/epd2.70035)
Supplement: Supplementary file 1 — Figure S1. [file EPD2-27-609-s001.pptx]

## Slide 1
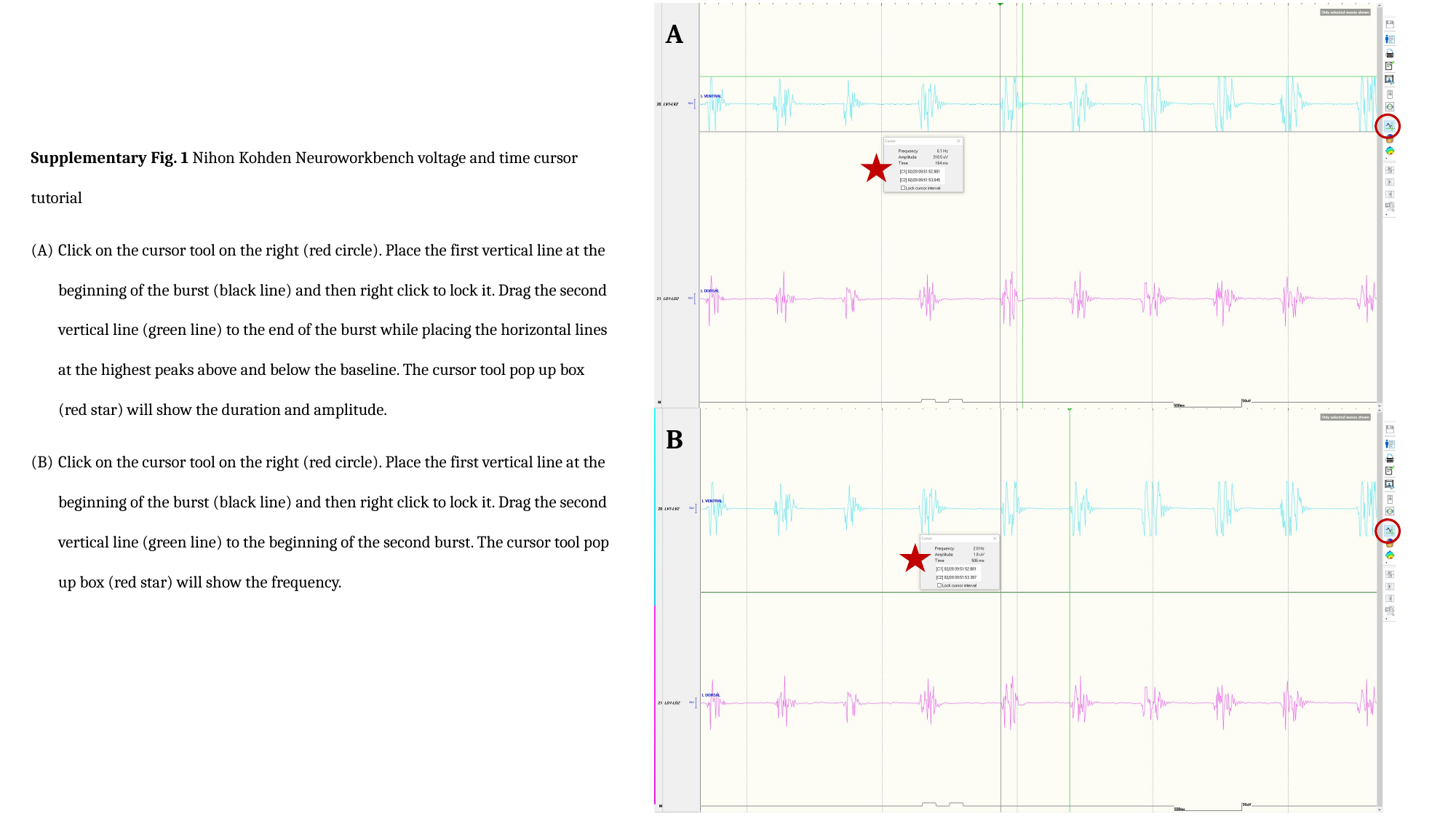

A
Supplementary Fig. 1 Nihon Kohden Neuroworkbench voltage and time cursor tutorial
Click on the cursor tool on the right (red circle). Place the first vertical line at the beginning of the burst (black line) and then right click to lock it. Drag the second vertical line (green line) to the end of the burst while placing the horizontal lines at the highest peaks above and below the baseline. The cursor tool pop up box (red star) will show the duration and amplitude.
Click on the cursor tool on the right (red circle). Place the first vertical line at the beginning of the burst (black line) and then right click to lock it. Drag the second vertical line (green line) to the beginning of the second burst. The cursor tool pop up box (red star) will show the frequency.
B
