# Supplementary material for: Surface‐electromyography characteristics of clonic seizures with no scalp‐EEG correlate: A comparative analysis with tremors
Source: Epileptic Disord. 2025 May 10;27(4):609–19. doi: 10.1002/epd2.70035 (PMC12398199; doi:10.1002/epd2.70035)
Supplement: Supplementary file 2 — Figure S2. [file EPD2-27-609-s005.pptx]

## Slide 1
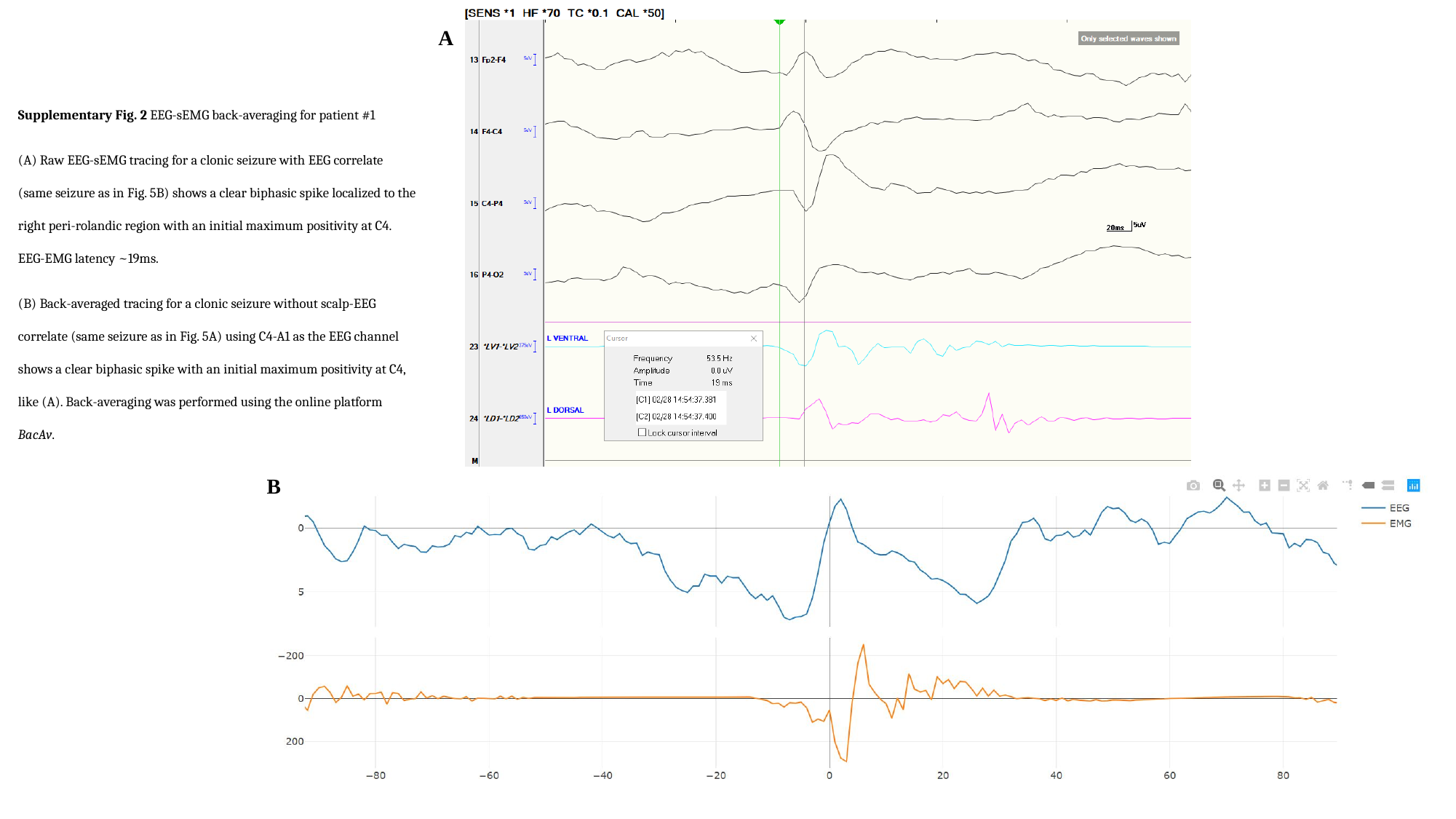

A
Supplementary Fig. 2 EEG-sEMG back-averaging for patient #1
(A) Raw EEG-sEMG tracing for a clonic seizure with EEG correlate (same seizure as in Fig. 5B) shows a clear biphasic spike localized to the right peri-rolandic region with an initial maximum positivity at C4. EEG-EMG latency ~19ms.
(B) Back-averaged tracing for a clonic seizure without scalp-EEG correlate (same seizure as in Fig. 5A) using C4-A1 as the EEG channel shows a clear biphasic spike with an initial maximum positivity at C4, like (A). Back-averaging was performed using the online platform BacAv.
B
