# Supplementary material for: Surface‐electromyography characteristics of clonic seizures with no scalp‐EEG correlate: A comparative analysis with tremors
Source: Epileptic Disord. 2025 May 10;27(4):609–19. doi: 10.1002/epd2.70035 (PMC12398199; doi:10.1002/epd2.70035)
Supplement: Supplementary file 3 — Figure S3. [file EPD2-27-609-s002.pptx]

## Slide 1
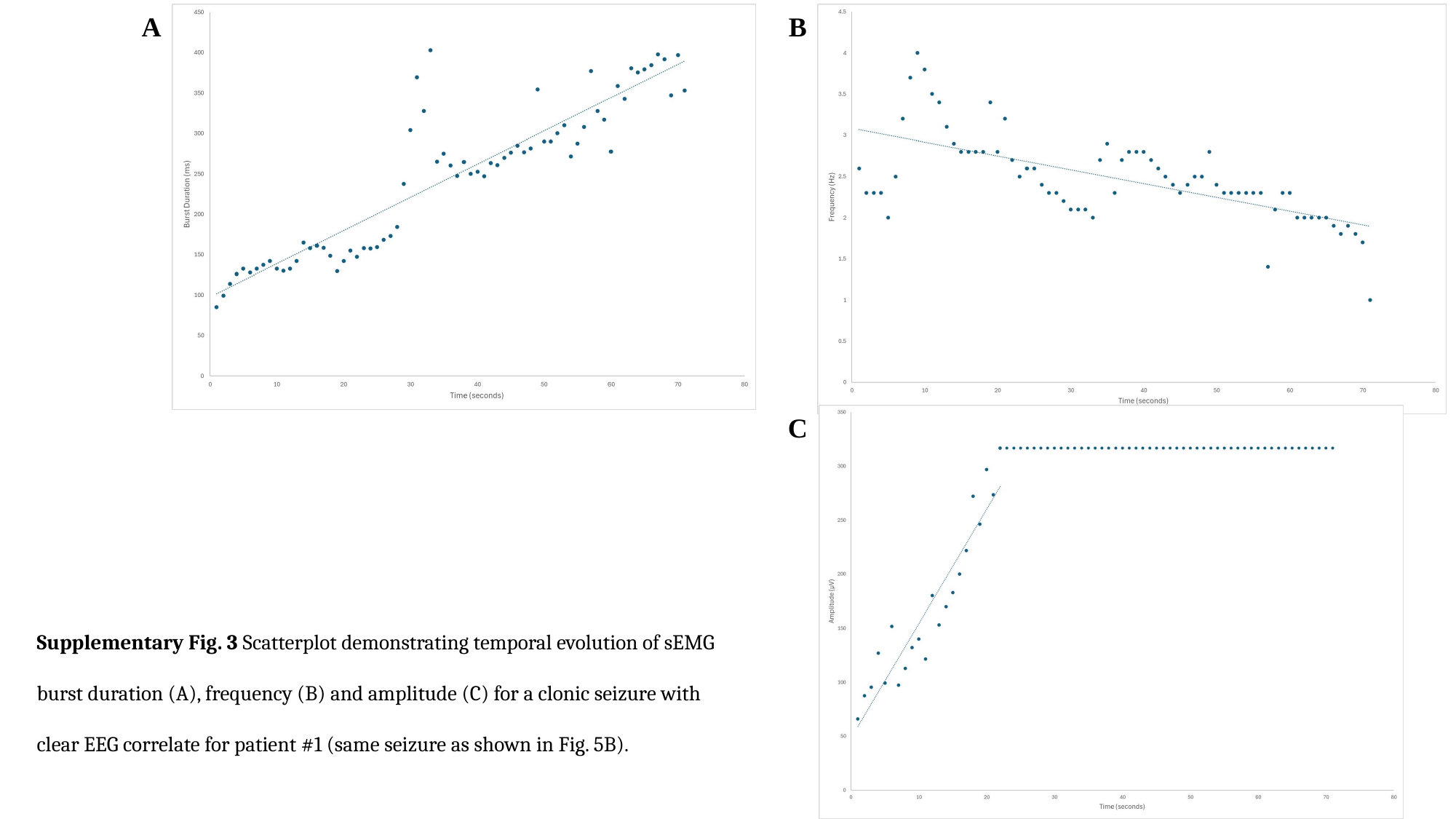

A
B
C
Supplementary Fig. 3 Scatterplot demonstrating temporal evolution of sEMG burst duration (A), frequency (B) and amplitude (C) for a clonic seizure with clear EEG correlate for patient #1 (same seizure as shown in Fig. 5B).
