# Supplementary material for: Surface‐electromyography characteristics of clonic seizures with no scalp‐EEG correlate: A comparative analysis with tremors
Source: Epileptic Disord. 2025 May 10;27(4):609–19. doi: 10.1002/epd2.70035 (PMC12398199; doi:10.1002/epd2.70035)
Supplement: Supplementary file 4 — Table S1. [file EPD2-27-609-s003.docx]

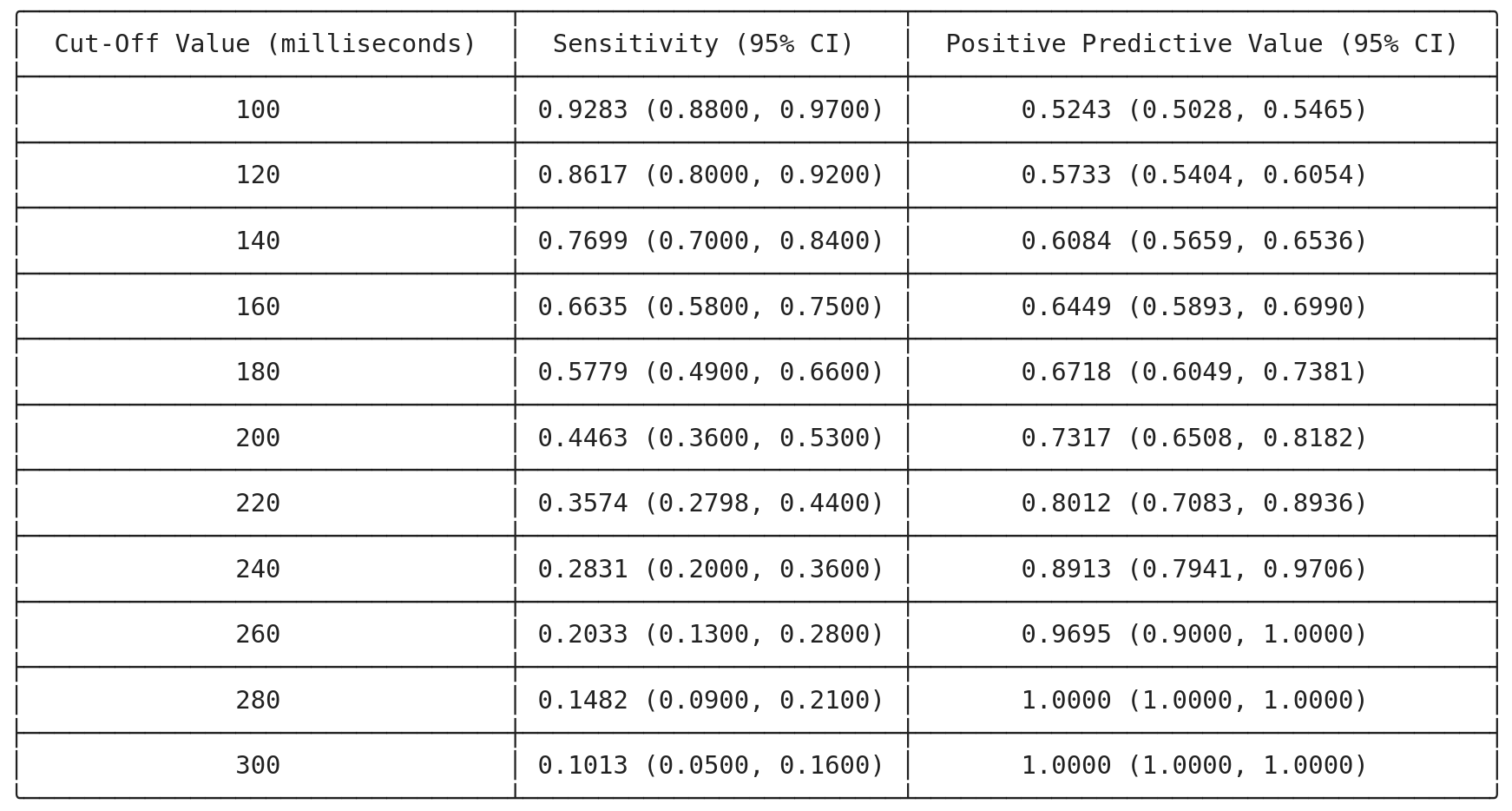


**Supplementary Table 1:** Sensitivities and Positive Predictive Values for sEMG burst durations to distinguish clonic seizures from tremors
