# Supplementary material for: Surface‐electromyography characteristics of clonic seizures with no scalp‐EEG correlate: A comparative analysis with tremors
Source: Epileptic Disord. 2025 May 10;27(4):609–19. doi: 10.1002/epd2.70035 (PMC12398199; doi:10.1002/epd2.70035)
Supplement: Supplementary file 5 — Appendix S1. [file EPD2-27-609-s004.docx]

**TEST YOURSELF**

**Answers:**

1. **D**

2. **D**

3. **B**
